# Surgical Management of Encapsulating Peritoneal Sclerosis: A Case Report in Kidney Transplant Patient

**DOI:** 10.1155/2018/4965459

**Published:** 2018-02-21

**Authors:** R. Shahbazov, M. Talanian, J. L. Alejo, F. Azari, A. Agarwal, K. L. Brayman

**Affiliations:** ^1^Department of Surgery, University of Virginia, Charlottesville, VA, USA; ^2^University of Virginia School of Medicine, Charlottesville, VA, USA

## Abstract

**Introduction:**

Encapsulating peritoneal sclerosis (EPS) is a clinical syndrome of progressive fibrotic change in response to prolonged, repetitive, and typically severe insult to the peritoneal mesothelium, often occurring in the setting of peritoneal dialysis (PD). Clear guidelines for successful management remain elusive. We describe the successful surgical management of EPS in a 28-year-old male s/p deceased donor kidney transplant for end-stage renal disease (ESRD) secondary to focal segmental glomerulosclerosis (FSGS). This patient received PD for 7 years but changed to hemodialysis (HD) in the year of transplant due to consistent signs and symptoms of underdialysis. EPS was visualized at the time of transplant. Despite successful renal transplantation, EPS progressed to cause small bowel obstruction (SBO) requiring PEG-J placement for enteral nutrition and gastric decompression. The patient subsequently developed a chronic gastrocutaneous fistula necessitating chronic TPN and multiple admissions for pain crises and bowel obstruction. He was elected to undergo surgical intervention due to deteriorating quality of life and failure to thrive. Surgical management included an exploratory laparotomy with extensive lysis of adhesions (LOA), repair of gastrocutaneous fistula, and end ileostomy with Hartmann's pouch. Postoperative imaging confirmed resolution of the SBO, and the patient was transitioned to NGT feeds and eventually only PO intake. He is continuing with PO nutrition, gaining weight, and free from dialysis.

**Conclusion:**

Surgical intervention with LOA and release of small intestine can be successful for definitive management of EPS in the proper setting. In cases such as this, where management with enteral nutrition fails secondary to ongoing obstructive episodes, surgical intervention can be pursued in the interest of preserving quality of life.

## 1. Introduction

Encapsulating peritoneal sclerosis (EPS) arises through a complex pathogenesis with numerous possible inciting factors [1]. EPS is characterized by marked inflammation and severe fibrosis of the peritoneum and is associated with high morbidity and mortality. It can occur years after termination of peritoneal dialysis (PD) and, in severe cases, leads to intestinal obstruction and ileus requiring surgical intervention [2]. The most common and well-characterized risk factor is peritoneal dialysis (PD), where EPS is the end result of progressive fibrotic change in response to prolonged, repetitive, and often severe insult to the peritoneal mesothelium [3, 4]. Healthy mesothelium is well adapted to cope with single episodes of peritonitis, demonstrating a robust ability to breakdown and resorb the inflammatory fibrous exudate left behind. Cyclic damage and gradual denudation of the mesothelium inhibits this capability [5]. Repeated serosal damage from the dialysate incites changes in the peritoneum. This results in a fibrous cap, trapping mesothelial stem cells and preventing them from reaching the surface; additionally, podoplanin and smooth muscle actin double-positive cells are thought to be involved in the pathogenesis of EPS [6]. PD is unique in terms of causative agents used and the degree and extent of sclerosis it incites [7]. Patients present with signs of deteriorating gastrointestinal function, obstruction, and loss of appetite [8]. Imaging or direct visualization in the proper clinical picture can aid in diagnosis.

Studies have demonstrated some variation in the incidence of EPS; however, they have agreed that the risk increases with time spent on PD. Clear guidelines for successful management remain elusive [9, 10]. Herein, we present a patient who developed multiple complications after kidney transplantation due to EPS and described its successful management.

## 2. Case Report

We report the successful surgical management of a 28-year-old male with EPS s/p kidney transplant in 2015 for end-stage renal disease (ESRD) secondary to focal segmental glomerulosclerosis (FSGS). Prior to transplant, the patient had been maintained on PD since 2009 but changed to hemodialysis (HD) in the spring of 2015 due to consistent signs and symptoms of underdialysis. EPS was visually identified at the time of transplant, and he did have an episode of emesis which he described as “green as grass” in the weeks leading up to transplant.

After transplant, his disease progressed to cause small bowel obstruction (SBO) requiring PEG-J placement for enteral nutrition and gastric decompression. This approach ultimately failed due to development of a chronic gastrocutaneous fistula necessitating chronic TPN, coupled with recurrent admissions for pain and bowel obstruction. After discussion with the patient, he was elected to undergo surgical intervention due to an increasingly poor quality of life and failure to thrive.

Surgical management included an exploratory laparotomy with extensive lysis of adhesions (LOA), repair of gastrocutaneous fistula, and end ileostomy with Hartmann's pouch. We began with a midline laparotomy, quickly encountering dense adhesions as expected. Next, we turned to the stomach, dissecting through unclear planes before ultimately taking down a portion of the greater omentum. Upon dissection anterior to the stomach, we found the location of the gastrocutaneous fistula, indicated by the audible suction sound coming from the NGT. The NGT was retracted, and the 5 cm defect closed with a TA60 stapler. The staple line was oversewn.

During extensive lysis of adhesions around the sigmoid colon, an enterotomy approximately 30% of the sigmoid circumference was made. Given the diffuse adhesions and relative immobility of the bowel, diversion with proximal ileostomy was elected. Following the procedure, he was sent to the floor with NGT in place and TPN. CT abdomen with contrast on postoperative day (POD) 6 showed resolution of the SBO. On POD 10, the NGT was withdrawn and trophic tube feeds plus clear liquid diet were tolerated well. TPN was withdrawn on POD 15, and the patient tolerated regular diet supplemented with tube feeds. Tube feeds were discontinued, and he was discharged home on POD 17.

Following discharge, he presented to OSH on POD 26 with acute kidney injury, high output from his ileostomy, and wound dehiscence. He was stabilized largely with aggressive rehydration and wound management and was discharged. Since then, the patient has been maintained on PO intake, avoiding sugary beverages to help keep ostomy output down. He has started gaining weight.

## 3. Discussion

It is generally thought that PD should be switched to HD at time of diagnosis of EPS; however, there are numerous factors that go into such a decision. Moreover, EPS has been noted to progress or present upon discontinuation of PD [11]. Pharmacotherapies for EPS including corticosteroids, tamoxifen, and immunosuppressants have been investigated with apparent lack of consistency [12]. The Pan-Thames study attempted to better assess these scattered reports, yet their cohort of 111 lacked sufficient power. They did not find any difference in survival between those treated with tamoxifen and/or immunosuppression when compared to no treatment. This is further supported by the fact that the Pan-Thames study and other authors observed many cases after transplant where patients were on large doses of immunosuppression [3, 13]. TPN has been a crucial of symptomatic management but carries risks and implications on quality of life. There is a growing body of evidence that surgical management is a useful tool when the situation demands definitive intervention, such as in our case [14, 15]. A multicenter Japanese study by Kawanishi et al. identified 48 PD patients that developed EPS (2.5%) and calculated both incidence and mortality over time and by intervention [16]. The incidence after 3, 5, 8, and >15 years was 0%, 0.7%, 2.1%, and 17.2%, respectively. The same study placed the overall mortality at 37.5%, increasing with time on PD [3]. Another group from Australia and New Zealand with a similar aim set the incidence at 0.3%, 0.8%, and 3.9%, at 3, 5, and 8 years, respectively [9].

We present this case as the successful surgical management of EPS. Our patient demonstrated many of the common findings of EPS: decreased efficacy of PD over time, characteristic changes observed upon laparotomy, obstructive symptoms, and onset of clinical symptoms following transplant. Management with enteral nutrition failed secondary to ongoing obstructive episodes, and TPN was required. The only solution we saw was definitive surgical intervention. Although some authors reported that perinectomy and enterolysis (PEEL) could be the first option for the management of EPS [15, 17], we believe that it may not relieve patient symptoms and could lead to more complications. Therefore, we choose extensive lysis of adhesion in our patient.

This case very much paralleled the series out of Tsuchiya General Hospital in Japan [16]. Those patients received similar management with laparotomy, enterolysis, and complete release of small intestine. The only repair of large intestine required was at the sigmoid colon, as in our case. Injury to the bowel was the most common complication. These were repaired; however, perforations led to sepsis and death in two of their patients [16]. Following the enterotomy in our case, we elected to divert with proximal ileostomy to protect the closure. This did not prevent the patient from developing infection; however, we do believe it was critical to his recovery.

## 4. Conclusions

Surgical intervention with LOA and release of small intestine has been demonstrated as a potential avenue for definitive management of EPS in the proper setting. The procedure is difficult and not without risk but can provide significant improvement in quality of life.

## Abbreviations

EPS: Encapsulating peritoneal sclerosis
PD: Peritoneal dialysis
ESRD: End-stage renal disease
HD: Hemodialysis
LOA: Lysis of adhesions
OSH: Outside hospital
SBO: Small bowel obstruction
TPN: Total parenteral nutrition
NGT: Nasogastric tube
FSGS: Focal segmental glomerulosclerosis
POD: Postoperative day.

## References

[1] Caicedo L., Delgado A., Caicedo L. A. (2017). Sclerosing encapsulated peritonitis: a devastating and infrequent disease complicating kidney transplantation, case report and literature review. *International Journal of Surgery Case Reports*.

[2] Reimold F. R., Braun N., Zsengeller Z. K. (2013). Transcriptional patterns in peritoneal tissue of encapsulating peritoneal sclerosis, a complication of chronic peritoneal dialysis. *PLoS One*.

[3] Kawanishi H., Kawaguchi Y., Fukui H. (2004). Encapsulating peritoneal sclerosis in Japan: a prospective, controlled, multicenter study. *American Journal of Kidney Diseases*.

[4] Yavuz R., Akbulut S., Babur M., Demircan F. (2015). Intestinal obstruction due to idiopathic sclerosing encapsulating peritonitis: a case report. *Iranian Red Crescent Medical Journal*.

[5] Tagnaouti M., Branger B., Ied C. (2009). Sclerosing encapsulating peritonitis: current features. *Nephrologie and Therapeutique*.

[6] Braun N., Alscher D. M., Fritz P. (2011). Podoplanin-positive cells are a hallmark of encapsulating peritoneal sclerosis. *Nephrology Dialysis Transplantation*.

[7] Machado N. O. (2016). Sclerosing encapsulating peritonitis: review. *Sultan Qaboos University Medical Journal*.

[8] Flood T. A., Veinot J. P. (2008). Test and teach. Diagnosis: sclerosing encapsulating peritonitis. *Pathology*.

[9] Johnson D. W., Cho Y., Livingston B. E. (2010). Encapsulating peritoneal sclerosis: incidence, predictors, and outcomes. *Kidney International*.

[10] Cestari A. T., Conti M. L., Prats J. A., Sato Junior H., Abensur H. (2013). Sclerosing encapsulating peritonitis after peritoneal dialysis. *Jornal Brasileiro de Nefrologia*.

[11] Brown E. A., Van Biesen W., Finkelstein F. O. (2009). Length of time on peritoneal dialysis and encapsulating peritoneal sclerosis: position paper for ISPD. *Peritoneal Dialysis International*.

[12] Huddam B., Azak A., Kocak G., Basaran M., Voyvoda N., Duranay M. (2012). Additive effectiveness of everolimus plus tamoxifen therapy in treatment of encapsulating peritoneal sclerosis. *Renal Failure*.

[13] Balasubramaniam G., Brown E. A., Davenport A. (2009). The Pan-Thames EPS study: treatment and outcomes of encapsulating peritoneal sclerosis. *Nephrology Dialysis Transplantation*.

[14] Tugcu M., Ruhi C., Boynuegri B. (2015). Successful ultrasound-guided percutaneous drainage of multiple splenic abscesses in a kidney transplant patient with encapsulated sclerosing peritonitis: a case report. *Transplantation Proceedings*.

[15] Latus J., Ulmer C., Fritz P. (2013). Encapsulating peritoneal sclerosis: a rare, serious but potentially curable complication of peritoneal dialysis-experience of a referral centre in Germany. *Nephrology Dialysis Transplantation*.

[16] Kawanishi H., Watanabe H., Moriishi M., Tsuchiya S. (2005). Successful surgical management of encapsulating peritoneal sclerosis. *Peritoneal Dialysis International*.

[17] Ulmer C., Braun N., Rieber F. (2013). Efficacy and morbidity of surgical therapy in late-stage encapsulating peritoneal sclerosis. *Surgery*.

